# Hospital Co-Operation in Hackney

**Published:** 1914-03-28

**Authors:** 


### Hospital Co-operation in Hackney.

A conference has been held at the Queen's Hospital for Children, Hackney Road, on the prevention or mitigation of summer diarrhœa amongst infants in the neighbourhood. The Earl of Shaftesbury, president of the hospital, took the chair, and in his opening remarks explained that the Conference had been called with the object of co-ordinating the various efforts that were now independently made. Lord Shaftesbury read a letter from the Local Government Board which had been sent to all the Metropolitan Borough Authorities, urging co-operation with voluntary organisations in regard to matters of public health.

Dr. Porter Parkinson, senior physician of the hospital, in moving the first resolution, stated that last year 870 cases had been brought to the hospital for treatment during the period of about six weeks commencing in the last week of August. It was an important part of the duty of the trained almoner, who had been employed by this hospital since 1903, to co-operate with other charities, and the making of arrangements for the "health visiting" of mothers with infants under out-patient treatment at the hospital takes up a great deal of her time. In the comparatively few cases that can be so visited under existing arrangements excellent results are obtained. He moved a resolution, which was carried unanimously, emphasising that epidemic summer diarrhœa is a preventable disease, which can best be dealt with by concerted action; and that Borough Councils and Boards of Guardians be urged to seek co-operation, with a view to arranging a complete system of home visiting in every case. Further, that all medical charities and all practitioners be urged to undertake the voluntary notification to the medical officer of health of all cases that may be brought to their notice.

On the motion of Mr. Joseph Meller, deputy-chairman of the house committee, a committee representing the Public Health Authorities, the Boards of Guardians, the hospital, and other charitable organisations of the neighbourhood was duly constituted, with Mr. T. Glenton-Kerr, secretary of the hospital, as hon. secretary.

